# Inducible somatic embryogenesis in *Theobroma cacao* achieved using the DEX-activatable transcription factor-glucocorticoid receptor fusion

**DOI:** 10.1007/s10529-017-2404-4

**Published:** 2017-07-31

**Authors:** Morgan E. Shires, Sergio L. Florez, Tina S. Lai, Wayne R. Curtis

**Affiliations:** Department of Chemical Engineering, The Pennsylvania State University, University Park, PA 16802-4400 USA

**Keywords:** Chimeric transcription factor, Chocolate, Glucocorticoid receptor, LEAFY COTYLEDON 2, Plant propagation, Somatic embryogenesis, *Theobroma cacao*

## Abstract

**Objectives:**

To carry out mass propagation of superior plants to improve agricultural and silvicultural production though advancements in plant cell totipotency, or the ability of differentiated somatic plant cells to regenerate an entire plant.

**Results:**

The first demonstration of a titratable control over somatic embryo formation in a commercially relevant plant, *Theobroma cacao* (Chocolate tree), was achieved using a dexamethasone activatable chimeric transcription factor. This four-fold enhancement in embryo production rate utilized a glucocorticoid receptor fused to an embryogenic transcription factor LEAFY COTYLEDON 2. Where previous *T. cacao* somatic embryogenesis has been restricted to dissected flower parts, this construct confers an unprecedented embryogenic potential to leaves.

**Conclusions:**

Activatable chimeric transcription factors provide a means for elucidating the regulatory cascade associated with plant somatic embryogenesis towards improving its use for somatic regeneration of transgenics and plant propagation.

**Electronic supplementary material:**

The online version of this article (doi:10.1007/s10529-017-2404-4) contains supplementary material, which is available to authorized users.

## Introduction

Asexual propagation (cloning) is a commercial practice used for a variety of reasons including superior plant clone performance, disease control, and necessity [for sterile plants such as banana and ornamental floral mutants; (Kumar and Reddy 2011; Tripathi et al. 2012)]. Asexual propagation ranges from proliferation of meristems, to highly differentiated somatic embryos (SE), which are anatomically similar to embryos found in seeds (Kamle et al. 2011). Since cloning is inherently more costly than seed production, its commercial potential depends on the individual plant value, propagation amplification ratio, and plant maturation rate. Orchids represent high individual plant value associated with clonal aesthetics; in 2005, 18 million orchids were sold in the US alone (Chugh et al. 2009). Progress towards synthetic seed generation using conifer somatic embryos by Weyerhaeuser (Gupta and Durzan 1987; Gupta and Hartle 2015) illustrates a combination of high clonal proliferation rates and superior plant performance, where individual plant value is relative low—and managing clonal diversity for long-term productivity is very important. Nestle has developed a scaled capacity of 3 million coffee somatic embryos per year (Ducos et al. 2007, 2010), that can provide plants at almost no cost to growers as a means of protection of this high-value plant product supply chain.

*Theobroma cacao* (Chocolate tree) was chosen for study of somatic embryogenesis because it generates high value products, relatively slow maturation (years), significant disease problems, genetic tools (genome, transformation) and has an established though low-efficiency SE process. In 2010, the chocolate industry was worth over 83 billion dollars (Report: MarketsandMarkets.com 2011). The majority of the world’s chocolate comes from cacao beans harvested in Latin America, Central Africa and Oceania, which experience at least 20% losses each year due to disease. The impact of production loss is particularly devastating to the predominant small, family-owned farms where disease can result in complete loss of income. We recently reported on the ability to utilize transient expression of the transcription factor BABY BOOM in tissues of *Theobroma cacao* to enhance the formation of somatic embryos (Florez et al. 2015b). This work complements our ongoing efforts to develop bioreactor propagation technology to allow for improved plant development by manipulating the physical environment (Florez et al. 2015a). In parallel with this previously reported transient gene expression method, which achieves transcription factor expression from *Agrobacterium* T-DNA that is not chromosomally integrated, we also sought to develop a highly-controlled inducible system based on stable transformation to avoid the complexity of *Agrobacterium* presence during TF expression. To decouple the expression of embryogenic transcription factor from its functional role of gene expression modulation, we adopted the DEX-activatable human glucocorticoid receptor fusion technology (Schena et al. 1991).

**Fig. 1:**
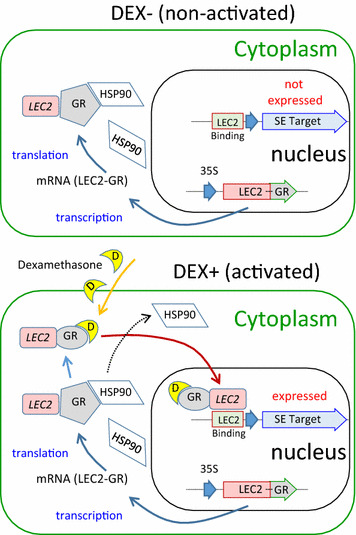
A schematic of the DEX glucocorticoid steroid activated transport of the transcription factor-glucocorticoid receptor fusion (i.e. LEC2-GR) to achieve an inducible and titratable means of manipulating somatic embryogenesis. The DEX (**D**) displaces the heat shock protein (HSP90) so that the transcription factor fusion can enter nucleus to activate its native gene targets. *Agrobacterium* genetic transformation introduces the constitutive 35s::LEC2(TF)-GR construct into the *T. cacao* genome

Induction of GR-fusion is controlled by the synthetic glucocorticoid dexamethasone (DEX). Due to its mammalian origin, DEX will bind to the mammalian GR with high specificity, thereby limiting off-target effects. This chimeric transcription factor system previously allowed for dose-dependent DEX activation of *LEC2* (Stone et al. 2008), based on constitutively expressing the GR fused to an *Arabidopsis* LEC2 transcription factor. In the absence of the DEX inducer, the native plant heat shock protein (HSP90) binds to the GR-fusion to prevent its transport into the nucleus (Fig. 1). Upon addition of DEX (**D**), HSP90 is quantitatively displaced from the complex to allow transport into the nucleus where its fused *LEC2* transcription factor domain can then mediate its associated regulatory function of inducing SE (Lutz et al. 2015). Stable transformation in *T. cacao* is an extremely rare event, requiring thousands of explants per transformant (Florez 2015). To overcome this limitation, visual screening using enhanced GFP (eGFP) was included to microscopically identify successful transformations. A constitutive promoter driving eGFP was included towards the left border of the T-DNA, where integration typically starts at the right border (Gelvin 2003), thereby increasing the chances of full T-DNA insertion and inclusion of the 35S:*TcLEC2*-*GR* element.

Initial performance of a single transformant based on the *T. cacao* TcLEC2 transcription factor are presented here. Experimentation on the transformed tissue demonstrates a titratable DEX dose-dependent enhancement in the quantity of somatic embryo formation. In addition, it was observed that somatic embryos could now be formed from explants of leaves of a regenerated 35S::*LEC2*-*GR* transgenic plant. The use of genetic transformation as a means to facilitate improved SE adds an interesting point of discussion for the role of genetically modified organisms (GMO) in the context of a high value commodity with a tremendous socioeconomic disparity among its stakeholders.

## Materials and methods

*Cacao/Agrobacterium*–*mediated transformations*: The procedure for transforming *T. cacao* var. Scavina-6 (SCA6) cacao somatic embryo cotyledons is detailed elsewhere (Florez et al. 2015b; Maximova et al. 2003), with specific conditions noted here. *A. tumefaciens* strain AGL1 harboring the desired plasmid was grown to an OD_600_ of 1 with activation using 100 µM acetosyringone. The co-cultivation time with *A. tumefaciens* on the filter paper was 4 days. Identification of the transgenic was accomplished by screening the developing genticin resistant (50 mg/l) somatic embryos for 15 weeks with visual checks for eGFP expression under a dissecting microscope to assess stable integration of the T-DNA region (after loss of the initial burst of ectopic transient eGFP expression). The *T. cacao LEC2* ortholog has been characterized by the Guiltinan laboratory (Zhang et al. 2014), and the *BABY BOOM* transcription factor (*TcBBM*) was cloned and characterized in Curtis laboratory (Florez et al. 2015b). These genes were fused to the glucocorticoid receptor domain (Stone et al. 2008), cloned into the *Spe*I and *Hpa*III sites of the pG00126 binary vector (Maximova et al. 2003), and transformed into the *Agrobacterium* strain AGL1 using standard molecular biology procedures.

*qPCR for Embryogenic Transcription Factors*: Fifty tissue explants were cut from the cotyledons of LEC2-GR embryos and then placed on solid ED media plates [Supplementary Table 4, (Guiltinan and Maximova 2010)]. Each cotyledon was cut into two tissue explants. One explant from each cotyledon was placed on solid ED media with a 50 µM DEX treatment and the other explant was placed on the negative ‘No DEX’ solid ED media control. Samples were collected 48-h after DEX treatment, snap frozen in liquid nitrogen and then stored in −80 °C. mRNA was extracted in triplicate biological replicates after grinding with liquid nitrogen using the Plant RNA reagent from Life Technologies per the manufacturer’s protocol. Total cacao RNA (1 µg) was treated with RNase-free DNaseI (NEB) to remove potential genomic DNA contamination, then reverse transcribed by ProtoScript II Reverse Transcriptase (NEB) with OligoDt(16mer) primers.

*qRT*-*PCR:* qRT-PCR was performed on diluted cDNA using SYBR Green Premix Ex Taq (Clonetech) scaled to10 μl using the Biosystem StepOne Plus Realtime PCR system under the following program: 15 min at 94 °C, 40 cycle of 15 s at 94 °C, 20 s at 60 °C, and 40 s at 72 °C. Primers (Supplementary Table 1) were designed to detect *TcLEC2*, *TcAGL15*, *TcBBM*, *TcLEC1*, and *TcFUS3* genes based on the coding sequence (Argout et al. 2011). A primer was also designed for the *LEC2*-*GR* mRNA that did not include the native *LEC2* mRNA noting that the native *LEC2* mRNA was expected to be in very low abundance relative to the constitutively expressed 35S::*LEC2*-*GR* fusion. The specificity of each primer pair was examined by PCR visualized on a 2% agarose gel and dissociation curve. The *Acyl Carrier Protein* (TcACP1), and a *Tubulin* gene in cacao (TcTUB1) were used as the reference genes. Statistical assessment for the effect of DEX addition was assessed using Student *T* test assuming equal variances.

*DEX dose response*: Cotyledons from secondary LEC2-GR embryos were cut and randomly distributed onto (10) plates of solid ED media with (10) embryos each. DEX (using 2 plates each) was investigated from 0 to 50 μM. After two weeks, all tissues were switched to solid ED media with no added plant hormones. Embryo formation on each DEX-containing media were counted and placed on fresh media every two weeks.

*Generating embryos from LEC2 DEX leaf*: A mature LEC2-GR embryo was converted to a greenhouse plant as described previously (Li et al. 1998). Briefly, mature embryos with developed cotyledon and axis were transferred to PEC media (Supplementary Table 5) (16/8 h photoperiod, 26–28 °C) and transferred to fresh media every four weeks. Once two true leaves were observed, the embryos were transferred to RD medium (Supplementary Table 6) refreshed monthly in a vented GA7 vessel. When primary and secondary roots were produced, the plant was then transferred to a D40 Deepot (Hummert) in commercial grade sand and placed on a misting bench (10 s mist/10 min) at light level of ~100 μE/m^2^s and fertilized with 1/10 Hoagland’s solution. Newly emerged leaves were cut from the greenhouse grown LEC2-GR transformed and non-transformed trees. The leaves were sterilized with 10% (v/v) bleach for 2 min followed by five 2-min washes in sterile water. Leaves were cut into ~2 mm square pieces (grouped top, middle and bottom), and successively placed for 2 weeks on solid PCG (Supplementary Table 2) and E5B media (Supplementary Table 3). Leaf explants were then transferred and maintained on solid ED media containing 10 µM DEX refreshed biweekly to observe embryo formation.

## Results

**Fig. 2:**
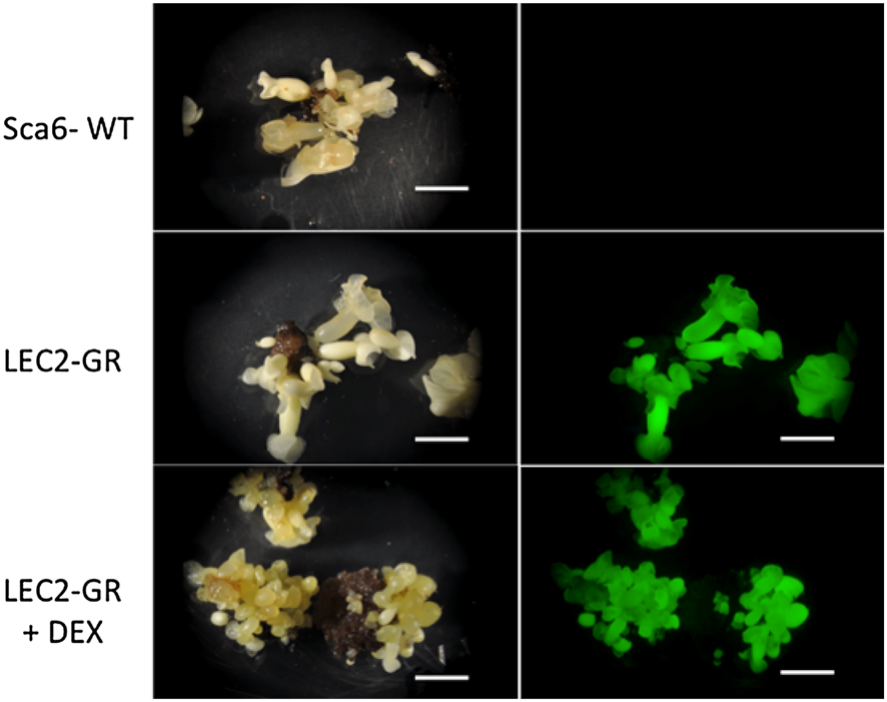
Expression of enhanced GFP in transgenic Lec2-GR *T. cacao* somatic embryos. Exposure to DEX resulted in enhanced SE proliferation

Cotyledon tissues of SCA6 were exposed to *Agrobacterium* containing binary vectors harboring the glucocorticoid fusions with BABY BOOM (*TcBBM*-*GR*) and LEAFY COTYLEDON 2 (*TcLEC2*-*GR*), on the T-DNA in tandem with eGFP followed by curing of *Agrobacterium* using 500 mg moxalactam/l plates as confirmed by lack of bacterial growth on R2A media (van der Linde 1999). Explants were then screened and monitored for eGFP fluorescence over a period of about 6 months. Consistent with strong recalcitrance to transformation, no fluorescing transformants of eGFP were observed for the* TcBBM*-*GR* transformation attempts on ~100 explants and 500 + secondary embryos; a single fluorescent, genticin-resistant transformed embryo was recovered from the comparable transformation with the *LEC2*-*GR* construct. The primary *LEC2*-*GR* transformant was readily proliferated by dissecting the cotyledons to produce large numbers of clonal transformed somatic embryos. Fluorescence of eGFP was uniform throughout these secondary embryos and constitutively expressed in DEX-treated or untreated cultures (Fig. 2). Exposure to DEX resulted in a notably enhanced proliferation of more numerous, though smaller, somatic embryos. This suggests the presence of a functional system where the introduction of DEX facilitates transport of the LEC2-GR fusion into the nucleus, thus implying the successful transformation of the cacao tissue.

**Fig. 3:**
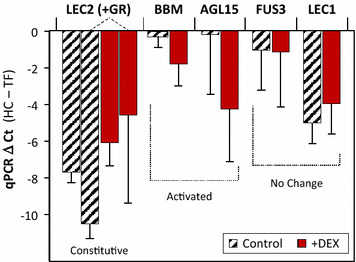
qPCR confirmation of highly expressed LEC2 mRNA (using total [native LEC2 plus LEC2+GR] and transgene specific [LEC2+GR] primers), and associated DEX activation of both the BABY BOOM (TcBBM) and AGAMOUS LIKE (TcAGL15) embryogenic transcription factors. The transcription factors LEC1 and FUS3 did not display differential activation upon exposure to DEX

Molecular confirmation of LEC2-GR activation and functionality was tested by examining potential downstream targets of the LEC2 transcription factor. Due to the nature of this activatable GR system, constitutive expression of the LEC2-GR fusion precludes utilizing mRNA/qPCR of LEC2 as an indicator of functionality. Noting that Ct values are simply a kinetic assessment of the rate of PCR amplification of template, Ct values that are lower are indicative of more mRNA. qPCR quantification of mRNA isolated from tissue 48 h after exposure to DEX is shown in Fig. 3. As anticipated, the levels of expression of LEC2-GR were higher as a result of constitutive expression from the enhanced 35 s-CaMV promoter. Consistent with this observation, the LEC2-GR-specific qPCR primers were highly expressed but not substantially different from the expression levels determined with a general LEC2 qPCR primers, indicating that the transgenic LEC2-GR mRNA dominates the native transcription factor expression (Fig. 3). BABY BOOM (*TcBBM*, *p* = 0.069) and AGAMOUS-LIKE (*TcAGL15*, *p* = 0.087) displayed substantial induction in the presence of DEX, from essentially zero background mRNA levels in non-activated tissues. This indicates that when DEX is added, it enters the cell and competes with the binding of HSP90 to allow for transport of the LEC2-GR construct to the nucleus and activates the downstream gene targets associated with the somatic embryogenic cascade. The additional transcription factors LEC1 and FUS3 did not display enhanced expression with the addition of DEX (*p* > 0.2). This adds to the recent effective demonstrations of the utility of the DEX/GR-fusion technology which, in addition to BABY BOOM study in Arabidopsis (Passarinho et al. 2008), was recently used to study transcription factor regulatory cascade for floral development in rice (Khanday et al. 2016).

**Fig. 4:**
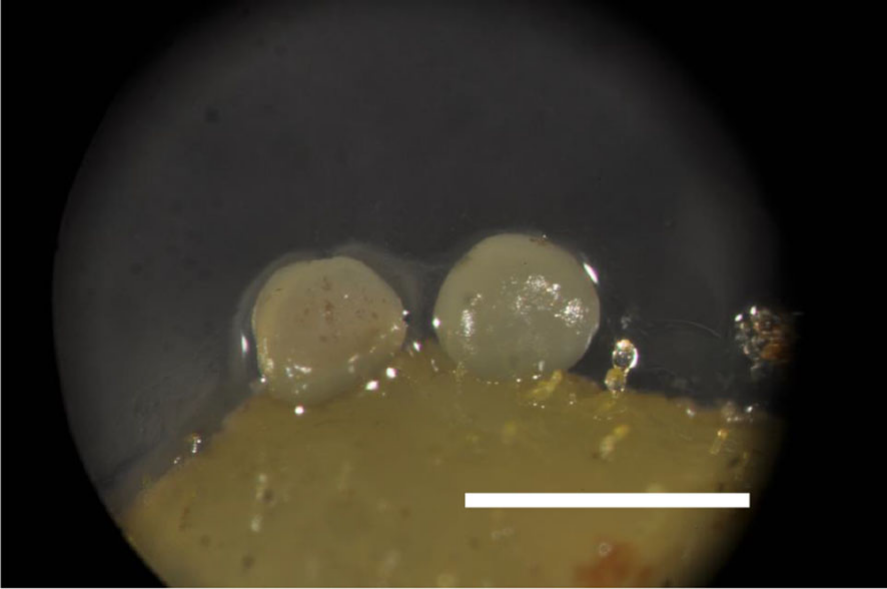
LEC2-GR tissue shows embryo formation after just 6 days. Embryo formation is observed (*white arrow*) after exposing the tissue to DEX (10 μM) for 6 days. *Scale bar* represents 1 mm

Upon confirming functionality of the LEC2-GR system at the molecular level, a simple test was undertaken to observe SE in the presence of DEX. Tissue of LEC2-GR secondary embryos was placed on hormone free-ED media supplemented with and without 10 μM DEX. After only 6 days, somatic embryos were observed in one of the DEX-treated explants (Fig. 4), while there was no embryo formation observed in the ‘no DEX’ control. This simple test motivated more extensive assessments of DEX-activated enhanced somatic embryo formation. In these small studies using dozens of embryos, there was consistent formation of multiple embryos on 10 μM DEX-treated LEC2-GR. Additionally, there were occasional observations of embryo formation from LEC2-GR cotyledon tissue on ED media without DEX treatment, suggesting there might also be some ‘leakiness’ of activation via transport occurring as well.

**Fig. 5:**
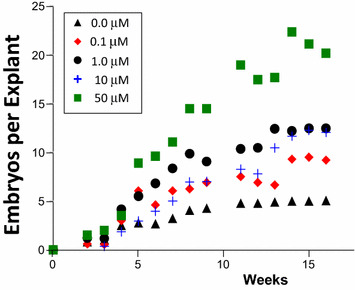
Dose response of LEC2-GR transgenic secondary embryo tissue to dexamethasone (DEX) treatments for transport-activated transcription factor enhancement of *T. cacao* somatic embryo formation

To provide a more definitive assessment of the functionality of the DEX/LEC2-GR system for enhancing somatic embryogenesis, a comprehensive dose response experiment was undertaken that involved 100 explants over five levels of DEX treatment observed over four months for accumulated SE production (Fig. 5). This experiment involved the production of 1077 LEC2-GR somatic embryos (403 at 50 µM) and provides a clear dose-dependent response for somatic embryo formation. The number of SE produced per explant for the highest DEX treatment (50 μM) was four-times higher than the untreated control. The 10 μM treatment was comparable to the 1 μM treatment; however, the 10 μM treatment included a plate of material that did not look as healthy as other tissue, emphasizing the need to work with relatively large numbers of tissue explants to overcome inherent variability that is common in plant tissue culture experimentation. A saturation of the dose response was not observed; therefore, it is not unlikely that even greater enhancements are possible.

**Fig. 6:**
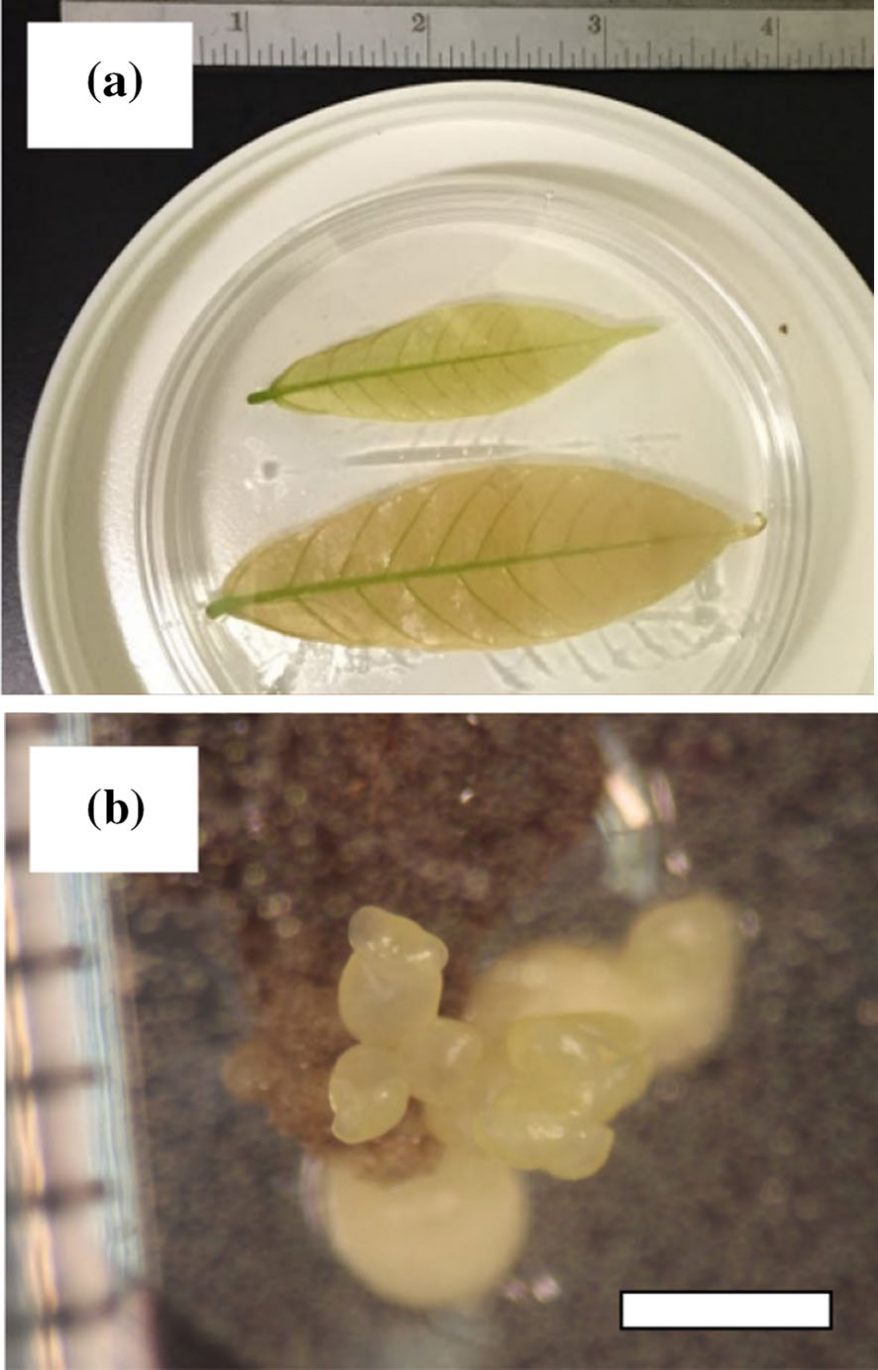
Induction of somatic embryos directly from the leaves of the TcLEC2-GR transgenic plant by surface sterilization and placement on DEX-containing ED media

During the execution of these experiments, a single mature somatic embryo was converted to a transgenic plant that was observed to develop normally (Supplementary Fig. 1). This regenerated transgenic plant was the first to develop primary leaves from less than a dozen of the most mature initial secondary somatic embryos, and was not part of a systematic study of conversion. From these young leaves, ~2700 mm^2^, tissue tested provided ~30 primary embryos over seven months with the first embryos appearing after three months. As a test of the more general ability of the DEX/LEC2-GR system to re-program plant development, a preliminary study was undertaken to examine if young leaves of this plant could provide a starting point for the production of primary somatic embryos. Like many plant species, *T. cacao* requires the use of specialized tissues to initiate somatic embryos; the standard procedure is to start with excised flower petals or stamens (Li et al. 1998). This ‘loss of juvenility’ is particularly pronounced for non-herbaceous plants, and is quite limiting in terms of sourcing plant tissue that can be used for studies of SE as well as genetic transformation. Surprisingly, we observed prolific somatic embryo formation on young LEC2-GR leaves that were surface sterilized and exposed to 10 μM DEX (Fig. 6) that did not take place for non-transgenic leaf controls. As young cacao seedlings only produce a flush of 2–4 leaves every few months, only two young leaves were sacrifices for this test.

As we move towards regeneration of secondary transgenics from the leaf-derived secondary embryos, there is a noticeable difference in the size of embryos. Leaf-derived LEC2-GR embryos are noticeably smaller than their progenitor secondary embryos (Supplementary Fig. 2). Nonetheless, these embryos are still quite large compared to the size of many other effective embryogenic systems we have worked with including carrot, Douglas fir, loblolly pine, and even red oak. Ongoing studies are examining methods for downstream conversion of these leaf-derived embryos in temporary immersion bioreactors.

## Discussion

Where many studies focus on rapid molecular regulatory events and fall short of executing longer term SE demonstrations, we have demonstrated that both *Agrobacterium*-based transient expression, and DEX-activated GR-fusion of embryogenic transcription factors have each provided enhanced production of cacao somatic embryos. Each approach has advantages and disadvantages for experimental and commercial implementation. The DEX/GR-TF approach is designed to avoid off-target effects (using a rat receptor component) and to avoid the stress and nuisance of *Agrobacterium* contamination. The need to create an initial transgenic plant can be very limiting, as demonstrated by our inability to obtain a *GR*-*BABY BOOM* transcription factor fusion despite considerable effort. The rate of transformation observed in this work was consistent with our prior selection of a 35S::BBM transformation that resulted in only one transformant from about 2000 screened explants (Florez et al. 2015b). We also contrast our prior conclusion that transient *Agrobacterium* expression of transcription factors with a constitutive promoter helps to prevent accidental generation of transformants because the constitutive expression of the TcBBM transcription factor resulted in aberrant proliferation and prevented regeneration.

These contrasting SE approaches provide an interesting context for the debate over GMO plants. The DEX/GR-TF approach to SE enhancement inherently requires the generation of a GMO, while the transient expression approach does not result in the integration of foreign DNA into the plant—though relies on similar DNA manipulations to create the transient vectors. Although SE “cloning” (i.e. clonal propagation) does not inherently generate a GMO, one can quickly see the potential for confusion based on the word association with molecular biology. Importantly, although non-GMO may be preferred by customers, it is questionable to impose the luxury of consumer preference onto either subsistence farmers, who could avoid catastrophic crop failure, or entire developing countries that require sufficient production for economic security. In the meantime, these tools show great promise for improving our understanding of SE while simultaneously advancing our capabilities for creating better crops (both GMO and non-GMO).

Although clonal propagation is not inherently a GMO technology, the need to regenerate plants from single-cell genetic transformation events (both for commercial varieties and scientific study) has been an important motivator towards technical advances in SE. Where rare events for SE are useful for plant genetic transformation, high efficiency SE is needed for commercial plant propagation. A single plant leaf contains millions of somatic cells; however, the process of SE relies on proliferating embryogenic plant tissues that are often derived from a specialized starting material such as immature embryos, gametophytic tissue, flower parts, or excised meristematic tissues (Elhiti et al. 2013; Fehér 2015). While hundreds of plant species have demonstrated this potential, it is often severely limited by species and even cultivar. The characterization of transcription factor cascades which lead to somatic embryo differentiation is rapidly being elucidated using advanced bioinformatic and molecular methods (Passarinho et al. 2008; Gliwicka et al. 2013). The utility of these early genetic markers must be confirmed with explicit demonstrations in improvements in SE. Therefore, improving our understanding of SE has the potential for greatly increasing plant propagation rates to expedite the introduction of higher productivity crop varieties—which is of increasing importance with rising world population.

In this work, we demonstrate not only that the heterologous GR fusion can function in *T. cacao* to facilitate dose-dependent activation of transcription factors, but also that it confers embryogenic potential to (young) leaf tissue. This improved embryogenicity is associated with the induced activation of a cascade of transcription factors associated with SE. The ability to expand the source tissue for SE is particularly notable for our propagation studies of *T. cacao*, where scaled-up bioreactor work is severely limited by the extensive tissue that is required for such experiments. The ability to rapidly generate tissue for bioreactor studies will permit a greater focus on elements of bioreactor design to see if our observations of oxygen-enhanced heterotrophic growth (Asplund and Curtis 2001), or CO_2_-mediated reduction in plant stress and enhanced growth in sugar-free media (Florez et al. 2015a) can be applied to economically-relevant species such as *T. cacao*. We are currently transitioning our efforts from this ‘luxury crop’ to food staples. Specially, we are working to develop propagation systems for African root crops such as yam, cassava and banana. The generality of transcription factors across the plant kingdom provides an opportunity to translate our findings from cacao to these and other plant species. The ability of the DEX-GR fusion approach to facilitate delivery of transcription factors to the nucleus also provides an elegant experimental system to elucidate the complexities of plant signaling in association with somatic embryo development.

## Electronic supplementary material

Below is the link to the electronic supplementary material.

Supplementary material 1 (PPTX 3221 kb)

Supplementary material 2 (DOCX 21 kb)

Supplementary material 3 (DOCX 50 kb)
