# Editorial Expression of Concern: Inducible somatic embryogenesis in *Theobroma cacao* achieved using the DEX-activatable transcription factor-glucocorticoid receptor fusion

**DOI:** 10.1007/s10529-023-03416-5

**Published:** 2023-08-01

**Authors:** Morgan E. Shires, Sergio L. Florez, Tina S. Lai, Wayne R. Curtis

**Affiliations:** Department of Chemical Engineering, The Pennsylvania State University, University Park, PA 16802-4400 USA

**Correction to: Biotechnology Letters (2017) 39:1747–1755** 10.1007/s10529-017-2404-4

The Editor-in-Chief is issuing an editorial expression of concern for this article. Concerns were raised regarding the methodology of this study including: inconsistencies between the methods and figures legends regarding the application of PGRs, three RNA extractions for the replicates from the same tissue rather than three extractions for different replicate cultures, and a lack of samples in Fig. 4 that would show that the embryogenesis would also not happen in non-transgenic explants. The authors were contacted and have been given an opportunity to provide a full response to these concerns. Sergio L. Florez, Tina S. Lai and Wayne R. Curtis have not responded to any correspondence from the editor/publisher about this editorial expression of concern. The editor was not able to obtain a current email address for Morgan E. Shires.

