# Supplementary material for: Inducible somatic embryogenesis in Theobroma cacao achieved using the DEX-activatable transcription factor-glucocorticoid receptor fusion
Source: Biotechnol Lett. 2017 Jul 31;39(11):1747–55. doi: 10.1007/s10529-017-2404-4 (PMC5636861; doi:10.1007/s10529-017-2404-4)
Supplement: Supplementary file 1 — Supplementary material 1 (PPTX 3221 kb) [file 10529_2017_2404_MOESM1_ESM.pptx]

## Slide 1
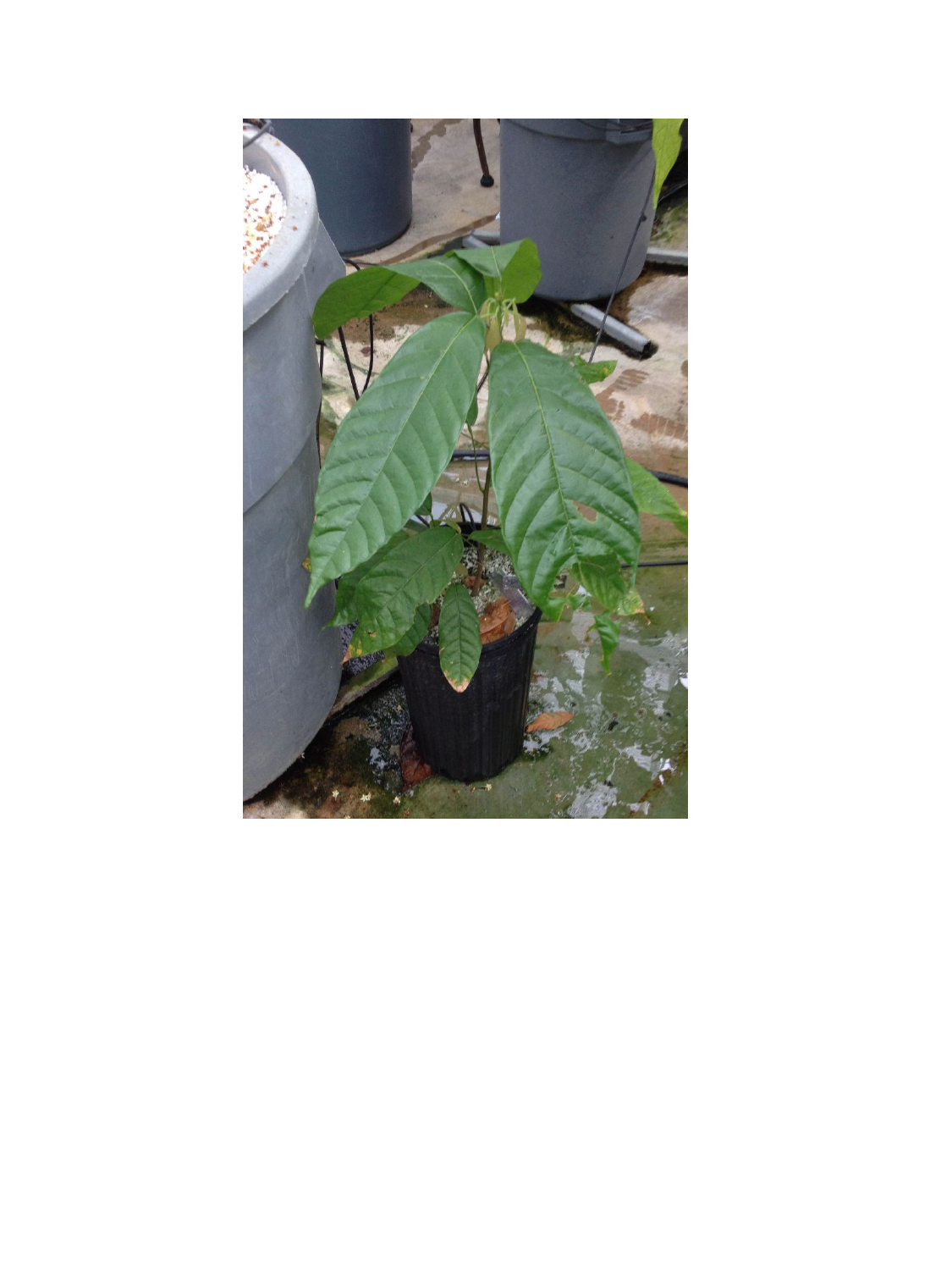

## Slide 2
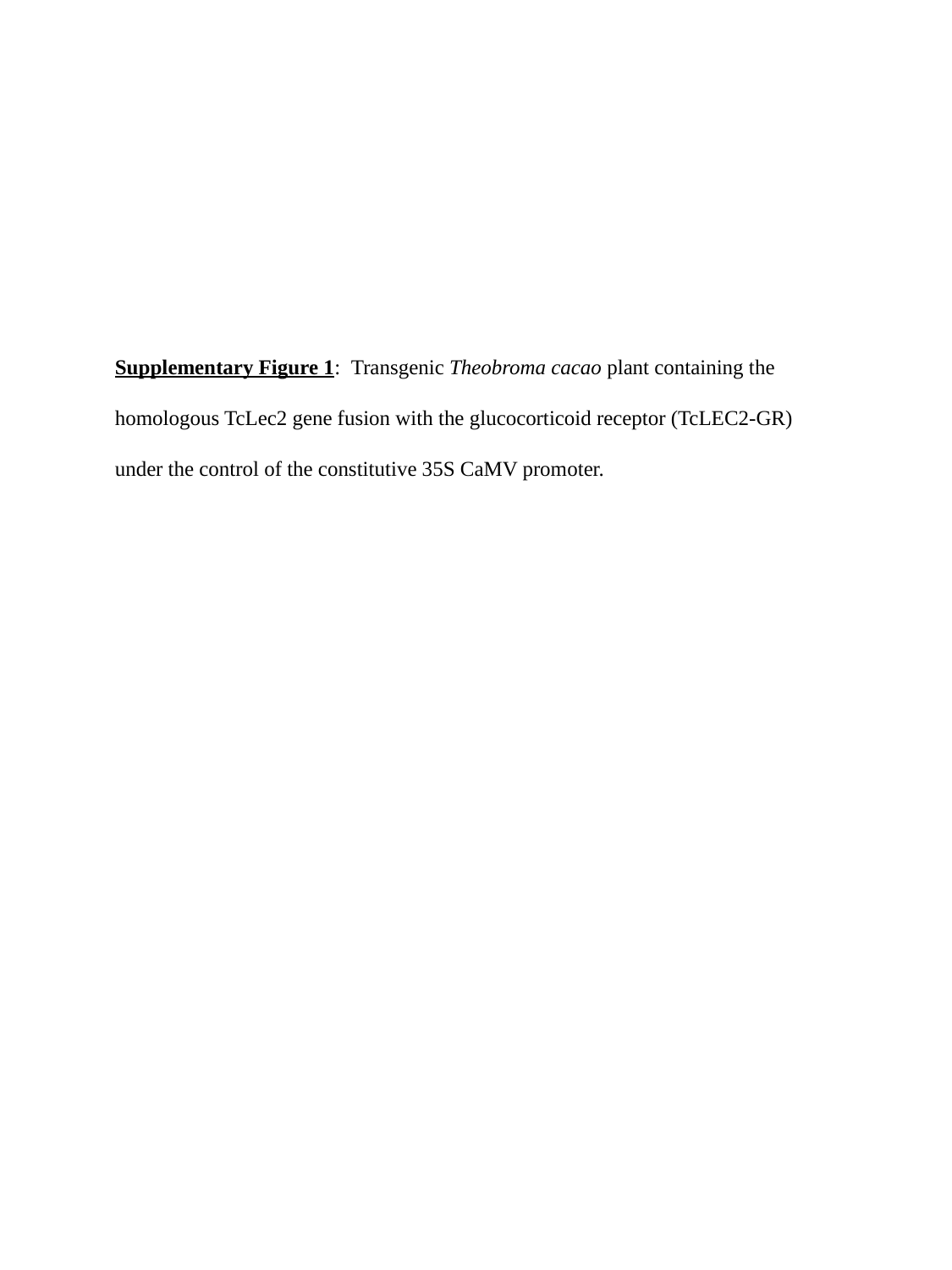

Supplementary Figure 1:  Transgenic Theobroma cacao plant containing the homologous TcLec2 gene fusion with the glucocorticoid receptor (TcLEC2-GR) under the control of the constitutive 35S CaMV promoter.

## Slide 3
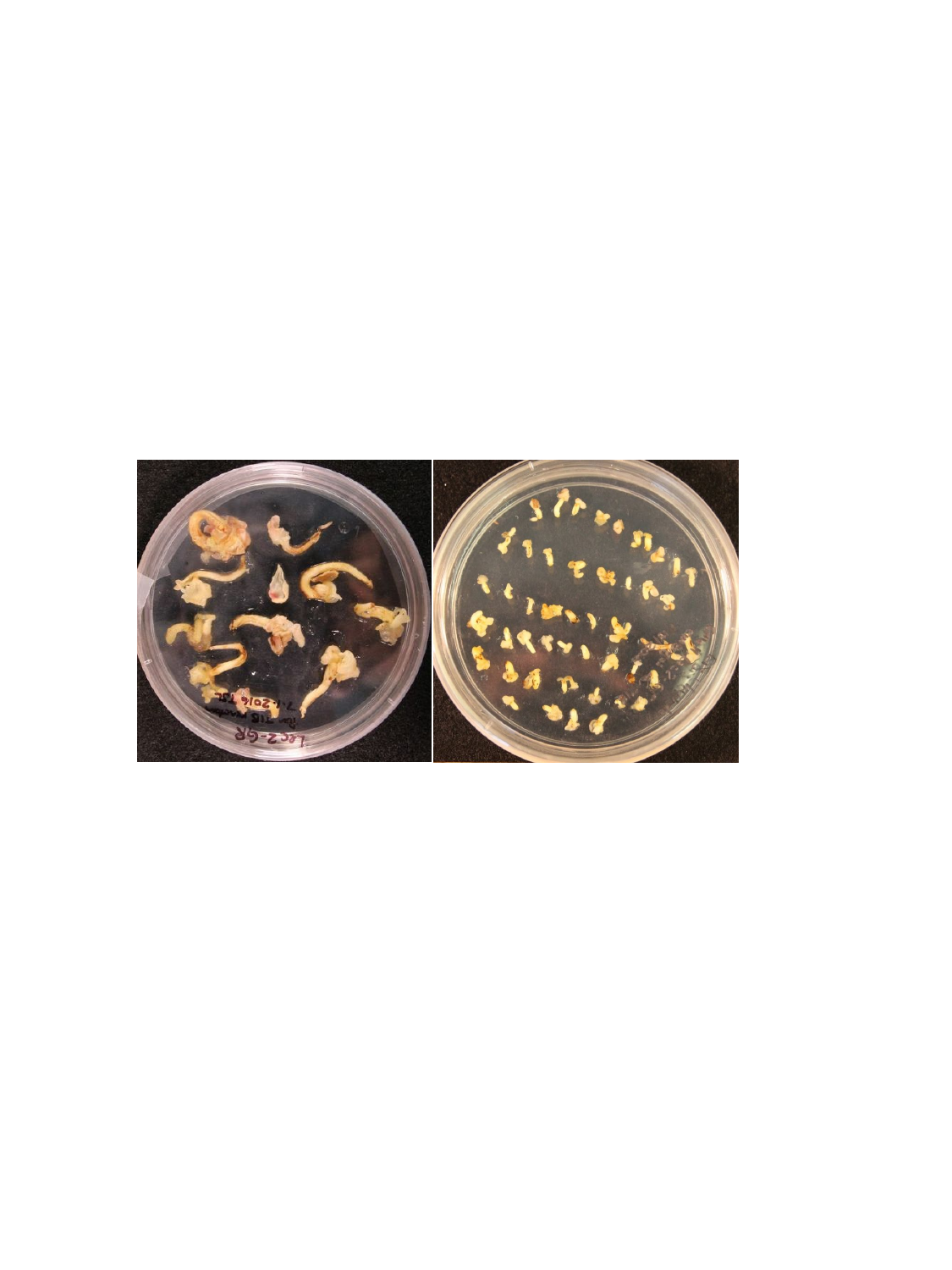

## Slide 4
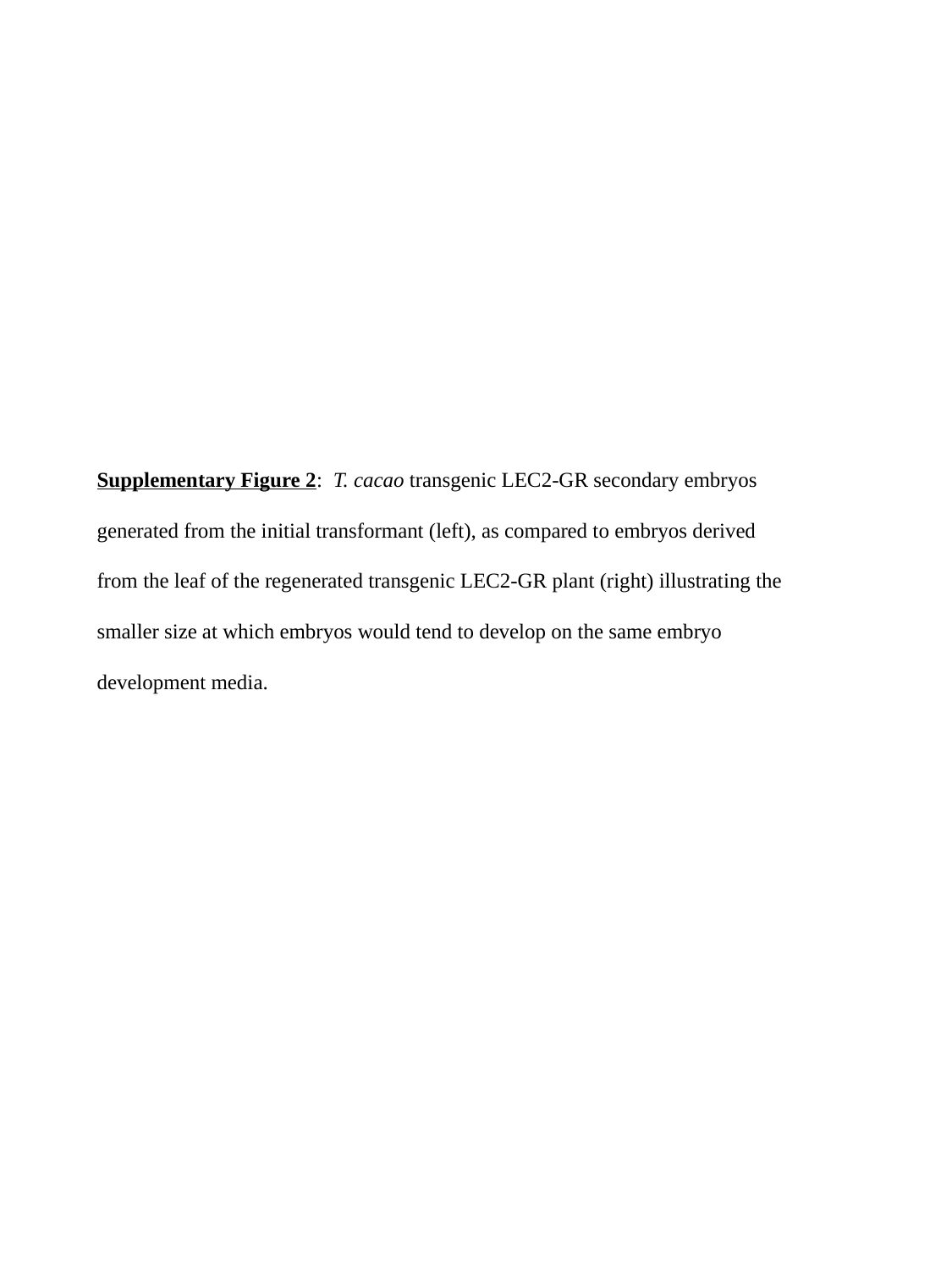

Supplementary Figure 2:  T. cacao transgenic LEC2-GR secondary embryos generated from the initial transformant (left), as compared to embryos derived from the leaf of the regenerated transgenic LEC2-GR plant (right) illustrating the smaller size at which embryos would tend to develop on the same embryo development media.
