# Supplementary material for: Inducible somatic embryogenesis in Theobroma cacao achieved using the DEX-activatable transcription factor-glucocorticoid receptor fusion
Source: Biotechnol Lett. 2017 Jul 31;39(11):1747–55. doi: 10.1007/s10529-017-2404-4 (PMC5636861; doi:10.1007/s10529-017-2404-4)
Supplement: Supplementary file 2 — Supplementary material 2 (DOCX 21 kb) [file 10529_2017_2404_MOESM2_ESM.docx]

Supplementary Table 1: qPCR Primer Sequences

| **Primer** | **Primer Sequence** | **Primer Description** | **Accession #** |
| --- | --- | --- | --- |
| LEC2GR-Forward | TGCTATGGCGGTCTTGATATG | Primers amplifying region between Glucocorticoid Receptor (GR) to beginning of the *Leafy-cotyledon 2 (LEC2)* gene | *-na-*  (transgene) |
| LEC2GR-Reverse | GCTTGCTGAATCCCTTTGATTT |  |  |
| ACP1-Forward | GGAAAGCAAGGGTGTCTCGTTGAA | Primers amplifying intron spanning region of housekeeping gene: *acyl-carrier protein-1* (ACP1) | *TcACP1*  *Tc01_p039970* |
| ACP1-Reverse | CCAAACAACAGCAGATTTCAACTCGC |  |  |
| BTUB-Forward | GGAGGAGTCTCTATAAGCTTGCAGTTGG | Primers amplifying intron spanning region of housekeeping gene: *beta-tubulin (*BTUB). | *TcTUB1*  *Tc06_p000360* |
| BTUB-Reverse | CTGGCTCTAGCTGGCTATGCTTATGT |  |  |
| AGL15-Forward | GGCAAGTCACATTCTCCAAGAGGC | Primers amplifying intron spanning region of *Agamous-15* *(AGL15)* gene. | *TcAGL15*  *Tc01_p040120* |
| AGL15-Reverse | CCAGAGCTGGCAAACTCAAAGAGC |  |  |
| BBM-Forward | GGTGCAAGCAGGAGCAAGATTCTG | Primers amplifying intron spanning region of *BabyBoom* *(*BBM*)* gene. | *TcBBM*  *Tc05_p019690* |
| BBM-Reverse | GAGCTATGCTCCATTGAAGAAGAGTCC |  |  |
| LEC1-Forward | ATGCAAAATCAAGCTCTGGGTTGAGC | Primers amplifying intron spanning region of *Leafy-Cotyledon 1* *(*LEC1*)* gene. | *TcLEC1*  *Tc07_p001180* |
| LEC1-Reverse | CATGGTGTTGGAGTTGGCGGT |  |  |
| LEC2-Forward | TGACCAGCTCTGGTGCTGACAATA | Primers amplifying intron spanning region of *Leafy-Cotyledon 2* *(*LEC2*)* gene. | TcLEC2  *Tc06_p015590* |
| LEC2-Reverse | ATTCTCCCAAGGGACCCAACATCA |  |  |
| FUS3-Forward | CTCCCCGCACGTGTTATTGATCC | Primers amplifying intron spanning region of *Fusca 3* *(FUS3)* gene. | *TcFUS3*  *Tc04_p004970* |
| FUS3-Reverse | GACATGCCAGTATCGTCCATCGTAGG |  |  |

**Additional Acknowledgments and Author Contributions**:

We would also like to specifically acknowledge assistance from the Guiltinan lab members Lena Sheaffer and Brian Rutowski for culture initiation and assistance with conversion of the 35S::LEC2-GR transgenic plant to the greenhouse. We also acknowledge Yufan Zhang for introduction of the TcLEC2 gene into the LEC2-GR *Agrobacterium* construct. S.F. and M.S. executed the dose response experiment. S.F. executed the transformation screen, identified and nurtured the transgenic *LEC2-GR* embryo to a plant.  M.S. maintained much of the tissue culture and executed the extended embryo dose response counting. T.L. assisted with cacao tissue culture proliferation and carried out the qPCR experiments. Support for cacao tissue culture work was also provided in the CurtisLab by Rachel Erwin and Ryan Jones with assistance in laboratory management and writing provided by Erica Lennox.
