# Supplementary material for: Inducible somatic embryogenesis in Theobroma cacao achieved using the DEX-activatable transcription factor-glucocorticoid receptor fusion
Source: Biotechnol Lett. 2017 Jul 31;39(11):1747–55. doi: 10.1007/s10529-017-2404-4 (PMC5636861; doi:10.1007/s10529-017-2404-4)
Supplement: Supplementary file 3 — Supplementary material 3 (DOCX 50 kb) [file 10529_2017_2404_MOESM3_ESM.docx]

Supplementary Table 2: Cacao (*Theobroma cacao*) Primary Callus Growth Medium (**PCG**)

|  | [final] | [stock] | prep / L | / 250 mL |
| --- | --- | --- | --- | --- |
| **Glucose** | -- | -- | **20 g** | **5 g** |
| **DKW macro A  (10x)** ^†^ |  |  | **100 mL** | **25 mL** |
| Ca (NO_3)2_∙4H_2_0 ^(A)^    MW=236.15 | 1.416 g/L | 14.16 g/L |  |  |
| NH_4_NO_3_(ammonium nitrate) | 1.969 g/L | 19.69 g/L |  |  |
| **DKW macro B  (10x)** |  |  | **100 mL** | **25 mL** |
| CaCl_2_•2H_2_O ^(A)^ | 0.149 g/L | 1.49 g/L |  |  |
| K_2_SO_4_ | 1.559 g/L | 15.59 g/L |  |  |
| MgSO_4_•7H_2_O ^(B)^ | 0.740 g/L | 7.40 g/L |  |  |
| KH_2_PO_4_ | 0.265 g/L | 2.65 g/L |  |  |
| **DKW micro (100x)** ^†^ |  |  | **10 mL** | **2.5 mL** |
| Zn(NO_3_)_2_**•**6H_2_O | 17.0 mg/L | 1.700 g/L |  |  |
| MnSO_4_**•**H_2_O ^(C)^ | 334.0 mg/L | 3.340 g/L |  |  |
| Cu SO_4_**•**5H_2_O ^(D)^ | 2.5 mg/L | 0.025 g/L |  |  |
| H_3_BO_3_ | 48.0 mg/L | 0.480 g/L |  |  |
| Na_2_MoO_4_**•**2H_2_O | 3.9 mg/L | 0.039 g/L |  |  |
| FeSO_4_**•**7H_2_O | 338.0 mg/L | 3.380 g/L |  |  |
| C_10_H_14_N_2_O_8_Na_2_**•**2H_2_O | 454.0 mg/L | 4.540 g/L |  |  |
| **DKW Vitamins (1000x)** **^ϔ^** |  |  | **1 mL** | **0.25 mL** |
| Thiamine-HCl | 2.0 mg/L | 2.0 g/L |  |  |
| Nicotinic acid | 1.0 mg/L | 1.0 g/L |  |  |
| Glycine | 2.0 mg/L | 2.0  g/L |  |  |
| **Inositol** ^(F)^ | 155.9 mg/L | 10 g/L (10 mg/mL) | **15.6 mL** | **3.9 mL** |
| **Glutamine** | 250 mg/L | 10.0 g/L | **25 mL** | **6.25 mL** |
| **2, 4-D**(ichlorophenoxyacetic acid) **^*^** | 2 mg/L | 0.1 mg/mL | **20 mL** | **5 mL** |
| **TDZ**(Thidiazuron) **^*^** | 5.0 µg/L | 0.2 mg/mL | **25 µL** | **6.25 µL** |
| pH = 5.8  (record initial pH) | | | | |
| 2.0 g/L Phytagel | | | **2 g** | **0.5 g** |

(A)   (dihydrate MW=147)  If using anhydrous CaCl_2_ (MW=111), stock contains 3.32 g/L

(B)   (heptahydrate MW=246.5)  If use anhydrous MgSO_4_ (MW=120),  stock contains 1.81 g/L

(C)   (manganese II sulfate, monohydrate MW=169.0) If use anhydrous MnSO_4_ (MW=151), stock contains 15.1 g/L.  Some older formulations MnSO_4_∙4H_2_O (MW=223.1), stock =22.3 g/L.

(D) (pentahydrate MW=249.7)  If use anhydrous CuSO_4_ (MW=159.6), stock = 0.016 g/L

(F) Inositol is stoichiometrically balanced to phosphate (6:1)

* 2, 4- D & TDZ can be added before autoclaving

**^ϔ^** Store aliquots of DKW vitamins in -20°C freezer; make fresh every 3-4 months

^†^ Stock solutions are stored at 4°C for no longer than 4 months

**Ref:**  Adapted from Guiltinan Lab’s *Integrated System for Vegetative Propagation of Cacao Protocol Book V. 2.1* (November 17, 2010)

Supplementary Table 3: Cacao (*Theobroma cacao*) Secondary Callus Growth Medium (**E5B**)

|  | [final] | [stock] | prep / L | / 250 mL |
| --- | --- | --- | --- | --- |
| **Glucose** | 20 g/L | -- | **20 g** | **5 g** |
| **WPS Macro A  (20x) ^†^** |  |  | **50 mL** | **12.5 mL** |
| Ca (NO_3)2_∙4H_2_0 ^(A)^    MW=236.15 | 0.4715 g/L | 9.43 g/L |  |  |
| NH_4_NO_3_(ammonium nitrate) | 0.4 g/L | 8.0 g/L |  |  |
| **WPS Macro B  (20x) ^†, *^** |  |  | **50 mL** | **12.5 mL** |
| CaCl_2_•2H_2_O ^(A)^ | 0.096 g/L | 1.92 g/L |  |  |
| K_2_SO_4_ | 0.990 g/L | 19.8 g/L |  |  |
| MgSO_4_•7H_2_O ^(B)^ | 0.344 g/L | 6.88 g/L |  |  |
| KH_2_PO_4_ | 0.170 g/L | 3.40 g/L |  |  |
| **WPS micro (100x)** ^†^ |  |  | **10 mL** | **2.5 mL** |
| Zinc Sulfate (ZnSO_4_•7H_2_O) | 8.6 mg/L | 0.86 g/L |  |  |
| Molybdic Acid (MoO_3_•H_2_O) | 0.25 mg/L | 0.025 g/L |  |  |
| MnSO_4_**•**H_2_O ^(C)^ | 22.3 mg/L | 2.23 g/L |  |  |
| Cu SO_4_**•**5H_2_O ^(D)^ | 0.25 mg/L | 0.025 g/L |  |  |
| H_3_BO_3_ | 6.20 mg/L | 0.620 g/L |  |  |
| FeSO_4_**•**7H_2_O | 27.8 mg/L | 2.78 g/L |  |  |
| **B5 Vitamins (1000x) ^ϔ^** |  |  | **1 mL** | **0.25 mL** |
| Thiamine-HCl | 10.0 mg/L | 10.0 g/L |  |  |
| Nicotinic acid | 1.0 mg/L | 1.0 g/L |  |  |
| Pyridoxine [HCl] ^(E)^ | 1.0  mg/L | 1.0  g/L |  |  |
| **Fe-EDTA 2H_2_O** (iron EDTA) ^(F)^ | 40 mg/L | 4.0 g/L | **10 mL** | **2.5 mL** |
| **Inositol ^(F)^** | 155.9 mg/L | 10 g/L (10 mg/mL) | **15.6 mL** | **3.9 mL** |
| **2, 4-D**(ichlorophenoxyacetic acid) ***** | 2 mg/L | 0.1 mg/mL | **20 mL** | **5 mL** |
| **BAP*** | 5.0 µg/L | 10 mg/mL | **5 µL** | **1.25 µL** |
| Record initial pH, Adjust pre-autoclave pH to 5.7 | | | | |
| Phytagel | | | **2.2 g** | **0.55 g** |

(A)   (dihydrate MW=147); If anhydrous CaCl_2_ (MW=111), stock = 1.45 g/L

(B)   (heptahydrate MW=246.5); If anhydrous MgSO_4_ (MW=120), stock = 3.361 g/L

(C)   (manganese II sulfate, monohydrate MW=169.0); If anhydrous MnSO_4_ (MW=151), stock = 15.1 g/L; If MnSO_4_∙4H_2_O, stock =22.3 g/L.

(D) (pentahydrate MW=249.7); If anhydrous CuSO_4_ (MW=159.6), stock = 0.016 g/L.

(E) (Ferrous EDTA: FeNaC_10_H_12_N_2_O_8_; anhydrous MW = 367.1) Original basis: 27.3 mg/L FeSO_4_ 7H_2_O (FW = 278) plus 37.3 mg/L Na_2_-EDTA (0.100 mM for both). Currently available iron-sodium-EDTA has a variable degree of hydration requiring Fe molarity equivalent calculation: (eg. Fe-EDTA [•2.5H_2_O] (FW=412.1), 4.12 g/L.

(E)   Pyroxidine MW is 169.18; however it appears to only be sold as pyroxidine HCl MW= 205.6

(F) Inositol is stoichiometrically balanced to phosphate (6:1 phytic acid)

* 2,4- D & BAP can be added before autoclaving

**^ϔ^** Store aliquots of DKW vitamins in -20°C freezer; make fresh every 3-4 months

^†^ Stock solutions are stored at 4°C for no longer than 4 months

**Ref:**  Adapted from Guiltinan Lab’s *Integrated System for Vegetative Propagation of Cacao Protocol Book V. 2.1* (November 17, 2010)

Supplementary Table 4: Cacao (*Theobroma cacao*) Embryo Development (**ED**) Medium

|  | [final] | [stock] | prep / L | / 250 mL |
| --- | --- | --- | --- | --- |
| **Glucose** | 1 g/L | -- | **1 g** | **0.25 g** |
| **Sucrose** | 30 g/L | -- | **30 g** | **7.5 g** |
| **DKW macro A  (10x) ^†^** |  |  | **100 mL** | **25 mL** |
| Ca (NO_3)2_∙4H_2_0 ^(A)^    MW=236.15 | 1.416 g/L | 14.16 g/L |  |  |
| NH_4_NO_3_(ammonium nitrate) | 1.969 g/L | 19.69 g/L |  |  |
| **DKW macro B  (10x) ^†^** |  |  | **100 mL** | **25 mL** |
| CaCl_2_•2H_2_O ^(A)^ | 0.149 g/L | 1.49 g/L |  |  |
| K_2_SO_4_ | 1.559 g/L | 15.59 g/L |  |  |
| MgSO_4_•7H_2_O ^(B)^ | 0.740 g/L | 7.40 g/L |  |  |
| KH_2_PO_4_ | 0.265 g/L | 2.65 g/L |  |  |
| **DKW micro (100x)** ^†^ |  |  | **10 mL** | **2.5 mL** |
| Zn(NO_3_)_2_**•**6H_2_O | 17.0 mg/L | 1.700 g/L |  |  |
| MnSO_4_**•**H_2_O ^(C)^ | 334.0 mg/L | 3.340 g/L |  |  |
| Cu SO_4_**•**5H_2_O ^(D)^ | 2.5 mg/L | 0.025 g/L |  |  |
| H_3_BO_3_ | 48.0 mg/L | 0.480 g/L |  |  |
| Na_2_MoO_4_**•**2H_2_O | 3.9 mg/L | 0.039 g/L |  |  |
| FeSO_4_**•**7H_2_O | 338.0 mg/L | 3.380 g/L |  |  |
| C_10_H_14_N_2_O_8_Na_2_**•**2H_2_O | 454.0 mg/L | 4.540 g/L |  |  |
| **DKW Vitamins (1000x) ^ϔ^** |  |  | **1 mL** | **0.25 mL** |
| Thiamine-HCl | 0.2 mg/L | 0.2 g/L |  |  |
| Nicotinic acid | 0.1 mg/L | 0.1 g/L |  |  |
| Pyridoxine [HCl] ^(E)^ | 0.2  mg/L | 0.2  g/L |  |  |
| **Inositol** ^(F)^ | 155.9 mg/L | 10 g/L (10 mg/mL) | **15.6 mL** | **3.9 mL** |
| Record initial pH, Adjust pre-autoclave pH to 5.7 | | | | |
| 2.0 g/L Phytagel | | | **2 g** | **0.5 g** |

(A)   (dihydrate MW=147)  If using anhydrous CaCl_2_ (MW=111), stock contains 3.32 g/L

(B)   (heptahydrate MW=246.5)  If use anhydrous MgSO_4_ (MW=120),  stock contains 1.81 g/L

(C)   (manganese II sulfate, monohydrate MW=169.0); If anhydrous MnSO_4_ (MW=151), stock = 15.1 g/L; If MnSO_4_∙4H_2_O, stock =22.3 g/L.

(D) (pentahydrate MW=249.7)  If use anhydrous CuSO_4_ (MW=159.6), stock contains 0.016 g/L.

(E)   Pyroxidine MW is 169.18; however it appears to only be sold as pyroxidine HCl MW= 205.6

(F) Inositol is stoichiometrically balanced to phosphate (6:1 phytic acid)

**^ϔ^** Store aliquots of DKW vitamins in -20°C freezer; make fresh every 3-4 months

^†^ Ensure that stock solutions are stored at 4°C for no longer than 4 months

**Ref:**  Adapted from Guiltinan Lab’s *Integrated System for Vegetative Propagation of Cacao Protocol Book V. 2.1* (November 17, 2010)

**For DEX Addition:**

|  | [final] | [stock] | prep / L | / 250 mL | / ~20 mL plate |
| --- | --- | --- | --- | --- | --- |
| **DEX** | 50 µM | 10 mM | 5 mL | 1.25 mL | 100 µL |
| **DEX** | 10 µM | 10 mM | 1 mL | 0.25 mL | 20 µL |

Supplementary Table 5: Primary Embryo Conversion (**PEC**) Medium

|  | [final] | [stock] | prep / L | / 250 mL |
| --- | --- | --- | --- | --- |
| **Glucose** | 20 g/L | -- | **20 g** | **5 g** |
| **Sucrose** | 10 g/L | -- | **10 g** | **2.5 g** |
| **Potassium Nitrate** | 0.3 g/L | -- | **0.3 g** | **0.075 g** |
| **DKW macro A  (10x) ^†^** |  |  | **100 mL** | **25 mL** |
| Ca (NO_3)2_∙4H_2_0 ^(A)^    MW=236.15 | 1.416 g/L | 14.16 g/L |  |  |
| NH_4_NO_3_(ammonium nitrate) | 1.969 g/L | 19.69 g/L |  |  |
| **DKW macro B  (10x) ^†^** |  |  | **100 mL** | **25 mL** |
| CaCl_2_•2H_2_O ^(A)^ | 0.149 g/L | 1.49 g/L |  |  |
| K_2_SO_4_ | 1.559 g/L | 15.59 g/L |  |  |
| MgSO_4_•7H_2_O ^(B)^ | 0.740 g/L | 7.40 g/L |  |  |
| KH_2_PO_4_ | 0.265 g/L | 2.65 g/L |  |  |
| **DKW micro (100x)** ^†^ |  |  | **10 mL** | **2.5 mL** |
| Zn(NO_3_)_2_**•**6H_2_O | 17.0 mg/L | 1.700 g/L |  |  |
| MnSO_4_**•**H_2_O ^(C)^ | 334 mg/L | 3.340 g/L |  |  |
| Cu SO_4_**•**5H_2_O ^(D)^ | 2.5 mg/L | 0.025 g/L |  |  |
| H_3_BO_3_ | 48.0 mg/L | 0.480 g/L |  |  |
| Na_2_MoO_4_**•**2H_2_O | 3.9 mg/L | 0.039 g/L |  |  |
| FeSO_4_**•**7H_2_O | 338 mg/L | 3.38 g/L |  |  |
| C_10_H_14_N_2_O_8_Na_2_**•**2H_2_O | 454 mg/L | 4.54 g/L |  |  |
| **DKW Vitamins (1000x) ^ϔ^** |  |  | **1 mL** | **0.25 mL** |
| Thiamine-HCl | 0.2 mg/L | 0.2 g/L |  |  |
| Nicotinic acid | 0.1 mg/L | 0.1 g/L |  |  |
| Pyridoxine [HCl] ^(E)^ | 0.2  mg/L | 0.2  g/L |  |  |
| **Inositol** | 155.9 mg/L | 10 g/L (10 mg/mL) | **39.4 mL** | **9.9 mL** |
| **Amino Acid Sol. (1000x) ^ϔ^** |  |  | **1 mL** | **0.25 mL** |
| Arginine |  | 43.55 mg/ 100 mL |  |  |
| Glycine |  | 18.76 mg/ 100 mL |  |  |
| Leucine |  | 32.80 mg/ 100 mL |  |  |
| Lysine |  | 45.65 mg/ 100 mL |  |  |
| Tryptophan |  | 51.05 mg/ 100 mL |  |  |
| Record initial pH, Adjust pre-autoclave pH to 5.8 | | | | |
| **Phytagel** | | | **1.75 g** | **0.44 g** |

(A)   (dihydrate MW=147); If anhydrous CaCl_2_ (MW=111), stock = 3.32 g/L

(B)   (heptahydrate MW=246.5); If anhydrous MgSO_4_ (MW=120),  stock = 1.81 g/L

(C)   (manganese II sulfate, monohydrate MW=169.0); If anhydrous MnSO_4_ (MW=151), stock = 15.1 g/L; If MnSO_4_∙4H_2_O, stock =22.3 g/L.

(D) (pentahydrate MW=249.7);  If anhydrous CuSO_4_ (MW=159.6), stock = 0.016 g/L.

(E)   Pyroxidine MW is 169.18; typically sold as pyroxidine HCl MW= 205.6 (ease of dissolution)

(F) Inositol is stoichiometrically balanced to phosphate (6:1 phytic acid)

**^ϔ^** Store aliquots in -20°C freezer; make fresh every 3-4 months; ^†^ refrigerate thawed stocks < 4 months

**Ref:**  Adapted from Guiltinan Lab’s *Integrated System for Vegetative Propagation of Cacao Protocol Book V. 2.1* (November 17, 2010)

Supplementary Table 6: Root Development (**RD**) Medium

|  | [final] | [stock] | prep / L | / 250 mL |
| --- | --- | --- | --- | --- |
| **Glucose** | 10 g/L | -- | **10 g** | **2.5 g** |
| **Sucrose** | 5 g/L | -- | **5 g** | **1.25 g** |
| **Potassium Nitrate** | 0.3 g/L | -- | **0.3 g** | **0.075 g** |
| **DKW macro A  (10x) ^†^** |  |  | **50 mL** | **12.5 mL** |
| Ca (NO_3)2_∙4H_2_0 ^(A)^    MW=236.15 | 1.416 g/L | 14.16 g/L |  |  |
| NH_4_NO_3_(ammonium nitrate) | 1.969 g/L | 19.69 g/L |  |  |
| **DKW macro B  (10x) ^†^** |  |  | **50 mL** | **12.5 mL** |
| CaCl_2_•2H_2_O ^(A)^ | 0.0745 g/L | 1.49 g/L |  |  |
| K_2_SO_4_ | 0.78 g/L | 15.59 g/L |  |  |
| MgSO_4_•7H_2_O ^(B)^ | 0.370 g/L | 7.40 g/L |  |  |
| KH_2_PO_4_ | 0.1325 g/L | 2.65 g/L |  |  |
| **DKW micro (100x)** ^†^ |  |  | **5 mL** | **1.25 mL** |
| Zn(NO_3_)_2_**•**6H_2_O | 8.5 mg/L | 1.700 g/L |  |  |
| MnSO_4_**•**H_2_O ^(C)^ | 167 mg/L | 3.340 g/L |  |  |
| Cu SO_4_**•**5H_2_O ^(D)^ | 1.25 mg/L | 0.025 g/L |  |  |
| H_3_BO_3_ | 24.0 mg/L | 0.480 g/L |  |  |
| Na_2_MoO_4_**•**2H_2_O | 1.95 mg/L | 0.039 g/L |  |  |
| FeSO_4_**•**7H_2_O | 169 mg/L | 3.38 g/L |  |  |
| C_10_H_14_N_2_O_8_Na_2_**•**2H_2_O | 227 mg/L | 4.54 g/L |  |  |
| **DKW Vitamins (1000x) ^ϔ^** |  |  | **0.5 mL** | **0.125 mL** |
| Thiamine-HCl | 0.1 mg/L | 0.2 g/L |  |  |
| Nicotinic acid | 0.05 mg/L | 0.1 g/L |  |  |
| Pyridoxine [HCl] ^(E)^ | 0.1  mg/L | 0.2  g/L |  |  |
| **Inositol** | 78.0 mg/L | 10 g/L (10 mg/mL) | **7.8 mL** | **1.95 mL** |
| Record initial pH, Adjust pre-autoclave pH to 5.8 | | | | |
| **Phytagel** | | | **1.75 g** | **0.44 g** |

(A)   (dihydrate MW=147); If anhydrous CaCl_2_ (MW=111), stock = 3.32 g/L

(B)   (heptahydrate MW=246.5); If anhydrous MgSO_4_ (MW=120),  stock = 1.81 g/L

(C)   (manganese II sulfate, monohydrate MW=169.0); If anhydrous MnSO_4_ (MW=151), stock = 15.1 g/L; If MnSO_4_∙4H_2_O, stock =22.3 g/L.

(D) (pentahydrate MW=249.7);  If anhydrous CuSO_4_ (MW=159.6), stock = 0.016 g/L.

(E)   Pyroxidine MW is 169.18; typically sold as pyroxidine HCl MW= 205.6 (ease of dissolution)

(F) Inositol is stoichiometrically balanced to phosphate (6:1 phytic acid)

**^ϔ^** Store aliquots in -20°C freezer; make fresh every 3-4 months; ^†^ refrigerate thawed stocks < 4 months

**Ref:**  Adapted from Guiltinan Lab’s *Integrated System for Vegetative Propagation of Cacao Protocol Book V. 2.1* (November 17, 2010)

**STOCK SOLUTION PREPARATION**:

**2, 4-D** =  2,4-Dichlorophenoxyacetic acid 0.1 mg/ mL stock

- - Dissolve 25 mg 2,4 -D in 0.75 mL 1 N NaOH in an epitube
  - Vortex until ~homogenous; put in water bath just until dissolved
  - Bring to volume in  250 mL volumetric flask  (**0.1 mg 2, 4-D / mL Stock**)
  - Distribute in 15 mL conical tubes and freeze (~indefinitely)
  - Use before 3 months at 4°C

**TDZ =** Thidiazuron = 1-Phenyl-3-(1,2,3-thiadiazol-5-yl)urea 0.2 mg/mL stock

- - Dissolve 1 mg TDZ in 1.0 mL 1 DMSO in an epitube
  - Bring to volume in  5 mL volumetric flask  (**0.2 mg TDZ / mL Stock**)
  - Aliquot into epitubes and freeze (~indefinitiely)
  - Use before 2 months stored at 4°C

**BAP** = BA =6-BAP (6-Benzylaminopurine) prep is dependent on form of BAP noting it has strange pK/dissociation behavior (and sold in multiple non-clarified / ambiguous forms which must be corrected for molecular weight). The hydrochloride salt will dissolve in either water or base (corrected for MW difference) the purine (base) form will dissolve in the 1N-HCl.

Example non salt form (MW=225.25; C_12_H_11_N_5_):

- - Dissolve 100 mg BAP in 3 mL 1 N KOH
  - Bring to volume in 10 mL volumetric flask  (**10 mg BAP / mL Stock**)
  - Distribute in epi-tubes and store frozen (~indefinitely)
  - Use before 3 months stored at 4°C

**DEX =** Dexamethasone 10 mM stock **(Light-Sensitive**)

- - Dissolve 0.03925 g DEX in 10 mL 200 proof ethanol in brown volumetric flask
  - Filter-sterilize
  - Aliquot into epitubes and freeze in opaque storage container
